# The Generation of Suspended Cell Wall Material May Limit the Effect of Ultrasound Technology in Some Varietal Wines

**DOI:** 10.3390/foods13091306

**Published:** 2024-04-24

**Authors:** Paula Pérez-Porras, Ana Belén Bautista-Ortín, Leticia Martínez-Lapuente, Zenaida Guadalupe, Belén Ayestarán, Encarna Gómez-Plaza

**Affiliations:** 1 Department of Food Science and Technology, Faculty of Veterinary Sciences, University of Murcia, 30071 Murcia, Spain; paula.perez2@um.es (P.P.-P.); anabel@um.es (A.B.B.-O.); 2 Institute of Vine and Wine Sciences, ICVV (University of La Rioja, Government of La Rioja and CSIC), Finca La Grajera, 26071 Logroño, Spain; leticia.martinez@unirioja.es (L.M.-L.); zenaida.guadalupe@unirioja.es (Z.G.); belen.ayestaran@unirioja.es (B.A.)

**Keywords:** polyphenols, polysaccharides, color, grape, ultrasound, enzyme

## Abstract

The disruptive effect exerted by high-power ultrasound on grape cell walls enhances phenolic extraction, improving chromatic characteristics during red wine maceration. However, short maceration times may, sometimes, hinder this enhancement, and this effect could be attributed to the suspended cell wall material formation facilitated by sonication. This suspended material, having a strong affinity for phenolic compounds, can lead to their precipitation and elimination during subsequent vinification stages and, consequently, a significant portion of extracted phenolic compounds may not contribute to the final phenolic composition of the wine, impacting its chromatic features. To demonstrate this effect, sonicated grapes of two different varieties were vinified with No modified process that eliminated part of this suspended material. Results confirm our hypothesis; that is, the lack of positive outcomes in some cases is due to phenolic compound adsorption on suspended material.

## 1. Introduction

Phenolic compounds are responsible for color and other sensory properties in wines. Due to their importance, their extraction from grape to must and the technologies that could facilitate this process have attracted a lot of attention for a long time, and very interesting reviews can be found [1,2]. Besides classic technologies such as maceration enzymes or run-off of the must, some new technologies designed to facilitate the extraction of compounds of interest in grapes, such as high-power ultrasound (US) and pulsed electric fields, have been approved by the OIV. These two technologies are non-thermal technologies and, therefore, they better maintain final wine quality than thermal technologies.

The extractability of the compounds from grape to must, since these compounds are located in the skin cells and surrounding by the cell walls, needs these cell walls to be dissembled before they can be extracted, and one of these new technologies, US, is very effective in doing so. By using this technology, we can either increase the extraction of phenolic compounds, compared to a similar vinification made from non-sonicated grapes [3], or reduce the maceration time needed to obtain a certain level of phenolic compounds in the final wine [4]. Although these studies have shown how, in general, US improved the extraction of phenolic compounds and the final chromatic characteristics of the wines—especially if long maceration times are used—this improvement has not been so evident in some other studies, especially with short maceration times [5].

Looking for an explanation, our previous research pointed to other factors that might contribute to this limited improvement. Among these factors, the interactions between phenolic compounds and the cell wall material suspended in the must from sonicated grapes could be included. These interactions exist, and have been described in the studies of Bindon et al. [6,7] for proanthocyanidins, and in the studies of Bautista-Ortín et al. [8,9] for proanthocyanidin and anthocyanins, and their occurrence in real vinifications have been demonstrated in wine samples by Osete-Alcaraz et al. [10,11].

Once the grapes are crushed to produce must for fermentation, the binding of phenolic compounds to this cell wall material may reduce their levels in the must, since these phenolic compounds may bind to the polysaccharides from skin and flesh cell walls (present at high concentrations at this moment) and finally be precipitated during subsequent steps of the vinification. The sonication, especially in varieties with thick cell walls, may increase the quantities of suspended material and escalate this problem. It is known that US increases the soluble polysaccharides in wines, which origin are grape cell walls, and that a varietal effect exists [3], and this is an indirect indication that US are clearly affecting grape cell walls dissembling and increasing the quantity of suspended material. 

To prove our hypothesis, we modified a traditional red wine vinification of sonicated grapes with a new step that includes the elimination of suspended material through enzymatic settling, prior to maceration and fermentation, so the role of the suspended material in the lower-than-expected phenolic compounds concentration in some wines made from sonicated grapes can be determined.

## 2. Materials and Methods

### 2.1. Wine Samples

Grapes of the red varieties of *Vitis vinifera* ‘Monastrell’ (Mo) and ‘Syrah’ (Sy) were harvested in September 2021 in Jumilla Appellation of origin area when grapes reached 25°Brix, which is considered the optimal moment of maturity for quality wines in this area. The grapes were immediately transported to the winery, where the clusters were destemmed and crushed. The initial must presented pH values of 4.0 and 3.79, a total acidity of 2.99 and 4.30 g/L of tartaric acid, and a total soluble content of 251.7 and 249.7 g/L, for ‘Monastrell’ and ‘Syrah’ musts, respectively. Four types of vinification were carried out in triplicate for each variety, using 45 kg of grapes for each vinification. These four types were a control vinification without US treatment (C), a vinification carried out with sonicated grapes (US), and two special vinifications: one control (CMV) and another with US treatment (USMV), which included a clarification or racking step similar to that used for white and rosé wines [12]. To carry out this process, the grapes were crushed (after which, for USMV processing, the US treatment was applied), and the skins were gently pressed (maximum pressure of 2 bars) in a 75 L membrane press. Must was treated with a pectolytic enzyme (3 mL/hL of Enozym Lux, Agrovin, Spain) and left to act at low temperature (10–12 °C) for 24 h. The pressed pomace was stored at 5 °C, and after 24 h, the clarified must was transferred to another tank and the pressed pomace was added. All the experiments were performed in triplicate.

The application of US was carried out using an industrial scale equipment (Ultrawine, Agrovin S.A, Alcázar de San Juan, Spain), applying a frequency of 30 kHz, a power of 9000 W, and a power density of 58.5 W/cm^2^. The design of the US equipment consists of two hexagonal sonoreactors with several adhere sonoplates, and it operates at a flow of 400 kg/h, allowing for the maintenance of the must temperature while the residence time inside the equipment is reduced.

All musts with the solid parts of the grapes were transferred to 50 L stainless steel tanks, where they underwent a 3-day fermentative maceration. The total acidity was corrected (5.5 g/L), and the yeast used was *Saccharomyces cerevisiae* (Viniferm CT007, Agrovin, Alcázar de San Juan, Spain) applied at a dose of 0.3 g/L. Fermentation was developed controlling the temperature at 27 °C. After 3 days of maceration, the solid parts of the grapes were pressed with a 75 L pneumatic press using a pressure of 2 bars. Once the fermentation was finished, the lees were discarded, and the wines were sulfited (0.07 g/L total SO_2_) and stabilized by cold for one month (2 °C). After that, they were racked again, sulfited and bottled, after which spectrophotometric and liquid and gas chromatography analyzes were carried out.

### 2.2. Wine Spectrophotometric Parameters

Analysis of chromatic parameters was carried out using a HEλIOS α spectrophotometer (ThermoSpectronic, Thermo Fisher Scientific, Madrid, Spain). The wine samples used in the analyzes were filtered using 0.45 µm nylon filters. The color intensity (CI) was calculated from the sum of the absorbances obtained at 420, 520 and 620 nm [13]. The determination of total and polymeric anthocyanins (TA and PA) was carried out following the method of Ho et al. [14]. The total polyphenol index (TPI) was determined at 280 nm according to the method of Ribéreau-Gayon et al. [15], and the analysis of methylcellulose precipitable tannins (MCPT) was determined at the same wavelength using the method proposed by Smith [16].

### 2.3. Determination of Tannins by HPLC

The concentration and tannic composition of the wines were determined using high-performance liquid chromatography (HPLC). Sample preparation was carried out according to the method proposed by Pastor del Río and Kennedy [17], with some modifications: 4 mL of wine filtered with 0.45 μm nylon filters were concentrated in a Centrivap equipment (Labconco, Kansas City, MO, USA). The concentrated wine was redissolved in 3 mL of HPLC quality water, and previously conditioned C18-SPE cartridges (1 g, Waters) (10 mL of methanol, 20 mL of HPLC quality water) were used to obtain the compounds of interest, these being eluted after washing the cartridge with 20 mL of HPLC-quality water using 10 mL of methanol, which were reconcentrated. The dry extract was redissolved in 400 μL of HPLC quality methanol, thus obtaining the methanolic extracts used in the phloroglucinolysis reaction, for which the process defined by Kennedy and Jones [18] was followed with some modifications: The phloroglucinolysis reagent was prepared with 100 g/L of phloroglucinol and 20 g/L of ascorbic acid in a solution of 0.2 N HCl in methanol. A total of 50 μL of the reagent and 50 μL of the methanolic extract were mixed and kept in a bath at 50 °C for 20 min, after which 100 μL of 200 mM sodium acetate was added to stop the depolymerization reaction and adduct formation. HPLC analysis followed the conditions described by Ducasse et al. [19]. A Waters 2695 system (Waters, Milford, MA, USA) equipped with a column Atlantis dC18 (250 × 4.6 mm, 5 μm packing) protected with a guard column of the same material (20 mm × 4.6 mm, 5 μm packing) (Waters, Milford, MA, USA) was used linked to a Waters 2996 photodiode array detector. The oven temperature was 30 °C. The injection volume was 10 μL and the flow rate 0.8 mL/min. Solvent A used for the elution was made of water/formic acid (98:2, *v*/*v*) (A), and solvent B was made of acetonitrile/solvent A (80:20 *v*/*v*) (B) [20,21].

Through the determination and quantification of adducts and flavan-3-ols, the concentration of total tannins (TT), the concentration of the subunits of epigallocatechin (EGC) and epicatechin gallate (ECG) and the percentage of galloylation (%Gal) were obtained, as well as the average degree of polymerization (mDP), calculated as the sum of all the subunits (flavan-3-ol monomer and phloroglucinol adducts, in moles) divided by the sum of all the flavan-3-ol monomers (in moles). 

### 2.4. Determination of Phenolic Compounds by SEC

The method proposed by Kennedy and Taylor [22] and adapted by Castro-López et al. [23] was developed for the determination of phenolic compounds by size exclusion chromatography (SEC). Samples were prepared from methanolic extracts obtained as specified in the previous section, and diluted with N,N-dimethylformamide (1/3 *v*/*v*).

The isocratic chromatographic method used a mobile phase composed of a solution of glacial acetic acid (1% *v*/*v*), MiliQ water (5% *v*/*v*), and lithium chloride (0.15 M) in N, N-dimethylformamide. A double column of PLgel (styrene-divinylbenzene copolymers in ethylbenzene; 300 × 7.5 mm each, 5 μm, with individual pore size of 100 and 500 Å, effective molecular mass range up to 4000 using polystyrene standards) was used protected by a guard column of the same material (50 × 7.5 mm, 5 μm), both provided by Polymer Labs (Amherst, MA, USA). The column was maintained at a controlled temperature of 60 °C. The injection volume was 10 μL and the flow rate used was 1 mL/min. Elution was monitored at 280 nm. Dimer and monomer procyanidins and skin and seed tannin fractions of known mean degrees of polymerization (mDP) were used as standards.

### 2.5. Identification and Quantification of Monosaccharides and Polysaccharides by GC–MS

Polysaccharides from the wine were obtained through precipitation following ethanolic dehydration, following the procedures described earlier [24,25]. To determine the monosaccharide composition, the *O*-methylglycosyl residues of trimethylsilyl ester produced after acid methanolysis and derivatization were subjected to GC-MS analysis [24,26,27,28], using an Agilent 7890A gas chromatograph (Agilent Technologies, Waldbronn, Germany) coupled to a 5975C VL quadrupole mass detector (MS). The total monosaccharide components of the precipitated polysaccharides (TMS) were determined. The content of mannoproteins (MP), rhamnogalacturonans type II (RG- II) and polysaccharides rich in arabinose and galactose (PRAG) was calculated as described previously [29], and the content of total soluble polysaccharide families (TPS) was estimated from the sum of PRAG, MP and RG-II [25,26].

### 2.6. Statistical Analyses 

Analyses of variance (ANOVAs) were performed using the SPSS v. 20.0 for Windows statistical package (SPSS Statistics, Inc., Chicago, IL, USA) program. Differences between means were compared using Tukey’s test. *p* < 0.05 was considered statistically significant.

## 3. Results and Discussion

### 3.1. Chromatic and Phenolic Composition of the Different Wines

Table 1 shows the chromatic characteristics of the studied ‘Monastrell’ and ‘Syrah’ wines.

In a previous study, where ‘Monastrell’ and ‘Syrah’ wines were made with sonicated grapes and seven days of skin maceration time, an increase in chromatic characteristics and phenolic content was clearly observed for both wines, although this increase was more important in ‘Syrah’ than ‘Monastrell’ wines [3]. In the present study, the analysis of the chromatic properties of ‘Monastrell’ wines made with three days of maceration (Table 1), and the tannin concentration (determined by phloroglucinolysis, Table 2) clearly showed that, with a maceration time of three days, and, contrary to what was expected, ‘Monastrell’ sonicated grapes led to a wine with lower color intensity, total phenol content, total anthocyanins and total tannins compared to the control wine. This result was surprising, since we could have expected a lack of positive results but never a decrease in the chromatic and phenolic values. 

The same results were not observed for ‘Syrah’ wines since, when ‘Syrah’ control wines were compared with ones made from sonicated grapes and three days of maceration, the color intensity increased from 19.40 to 23.47, and an increase of 17% and 25% in the values of total phenols and MCPT and 42% in the concentration of tannins could also be observed.

In the previously mentioned study [3], where a maceration time of seven days was used, the wines made from different varieties (‘Monastrell’, ‘Syrah’ and ‘Cabernet Sauvignon’) increased their chromatic characteristics when they were made with sonicated grapes. However, one interesting observation was that the percentage of increase caused by the application of US was varietal-dependent. We could observe how the application of US resulted in an increase in the total phenol content of 20% for ‘Syrah’ wine and only 6% for the ‘Monastrell’ wines. Similarly, the increase in MCPT was 32% for ‘Syrah’ and only 20% in ‘Monastrell’ wines. The study suggested that the extension of the cell wall disruption caused by US varied depending on grape skin cell wall composition and structure, and that the skin thicker cell walls of varieties such as ‘Monastrell’ were more difficulty degraded by US. In fact, ‘Monastrell’ grape skins, when studied by optical microscopy [3], showed more layers of skin cells and thicker walls than ‘Syrah’ grape skins, and ‘Cabernet Sauvignon’ skin cells also presented quite thick cell walls although, in general, their integrity seems to be more compromised. These morphological differences may also be confirmed by looking at the quantity and composition of isolated cell wall material in fresh grapes. After seven days of maceration, the skin structure was more disassembled when US was used, and this was especially significative in ‘Syrah’ and ‘Cabernet Sauvignon’ macerated skins.

However, in this experience, involving a three-day maceration, the unexpected effect of US was that it caused a decrease in the concentration of phenolic compounds and color intensity of ‘Monastrell’ wines, suggesting an additional phenomenon influencing the chromatic characteristics, and warranting further investigation.

Looking for an explanation of this unexpected result, a retrospective examination of prior investigations was undertaken, pointing to the role of suspended cell wall material in the absorption and, afterwards, a reduction in the phenolic concentration in wines. As Osete-Alcaraz et al. [11,12] stated, the interaction between the extracted phenolic compounds and the skin and pulp cell walls suspended in the must may constitute an important concern. The components of the cell walls show high affinity for phenolic compounds and adsorb them into their structure [6,7,8,9,23,30,31,32] and, therefore, after grape crushing and sonication, phenolic compounds are extracted, but a great amount of suspended cell wall material is also generated. The studies of Osete-Alcaraz et al. [10] demonstrated that the phenolic compounds bind to the suspended cell material and, once this binding has occurred, these interactions are difficult to reverse, and they may reduce the phenolic concentration in the must and the final wine, as the phenolic compounds bound to the cell walls precipitate with them during the following stages of vinification. When the quantity of the suspended material is very large, an important part of the extracted phenolic compounds will not become part of the final wine’s phenolic composition, affecting its chromatic characteristics. This effect could be important in all situations, but much more significant when varieties with thick cell walls (as ‘Monastrell’) are sonicated and vinified, and when short maceration times are used, since the maceration time for allowing a large extraction of phenolic compounds that could overcome the losses due to cell wall adsorption is limited.

To test the hypothesis, a modification of the winemaking process was carried out, and this modification included a partial elimination of suspended material through enzymatic settling at the beginning of the process, prior to maceration and fermentation, thus favoring the partial elimination of the suspended material. The main enzymatic activities of the enzyme used for the must settling, Enozym Lux, were (expressed as enzyme units, as provided by the supplier) endo-polygalacturonase (EC 3.2.1.15, 4500 U/g), pectin methyl esterase (EC 3.1.1.11, 1000 U/g) and pectin-lyase (PL) (EC4.2.2.10, 130 U/g). This enzyme has shown, in previous studies, to have a high capacity to degrade cell wall pectins from the skin and produce a large quantity of soluble low molecular weight polysaccharides [31,32]. Moreover, Osete-Alcaraz et al. [11] reported a low content of pectins (3.55 ± 0.64 mg/g of fiber cell wall) in the precipitated pulp fiber after using this enzyme in Monastrell must.

The results showed that the elimination of the suspended material slightly increased color intensity in ‘Monastrell’ control wine, and it largely did so in the vinification made from sonicated grapes, where color intensity increased by 36%, and important increases were also observed in TP and, especially, in TT. Regarding the same experience in ‘Syrah’ grapes, the modified vinification also increased chromatic characteristics but, compared to the effect on sonicated grapes, no differences were observed. It seems that sonication does not lead to as high a quantity of suspended material in ‘Syrah’ vinifications as in ‘Monastrell’. The phloroglucinolysis analysis in ‘Monastrell’ wines showed that tannin concentration in control wine made with the modified vinification did not significantly differ from control vinifications, although mDP slightly increased, but the tannin concentration did increase a lot when suspended material was eliminated from the elaboration with sonicated grapes, with their mDP also slightly increasing, which may be related to cell walls mainly binding high molecular mass tannins [33,34], so the limited presence of cell walls increased the mDP of the tannins. In ‘Syrah’, the modified vinification did not change the concentration of tannins, nor the mDP or % of EGC, indicating the less important role of suspended material in ‘Syrah’ vinifications, due to a lower presence of suspended cell wall material, and to ‘Syrah’ cell walls presenting less affinity for phenolic compounds than those of ‘Monastrell’ [32].

**Figure 1:**
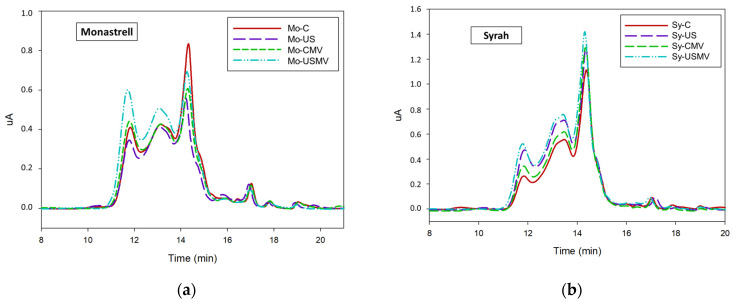
(**a**) Analysis of the phenolic profile of studied ‘Monastrell’ wines by size exclusion chromatography. (**b**) Analysis of the phenolic profile of ‘Syrah’ studied wines by size exclusion chromatography.

Figure 1a and 1b and Table 3 showed the molecular mass distribution of the phenolic compounds of the different wines obtained by Size Exclusion Chromatography (SEC).

Differences are clearly observed in the profiles of the different wines (Table 3). The removal of suspended material greatly increased the phenolic compounds of the high molecular mass (HMM) in the control wine made from Monastrell, and reduced those of lower molecular weight (LMM), which is consistent with the increase in mDP, while the sonicated sample showed an increase in all three fractions analyzed (Figure 1a). On the other hand, for Syrah wines (Figure 1b), the elimination of suspended material only resulted in very small differences in the phenolic profile compared to their wines elaborated with normal vinification and for both control and sonicated wines, with the difference due to the sonication of the grapes being more important in the modification in the profile than the elimination of suspended material. 

### 3.2. Polysaccharide Composition of the Different Wines

We were also interested in the effect of sonication, and this modification in the elaboration procedure on the polysaccharide composition of the wines. These compounds only arose from the degradation of cell walls originated during crushing so, their behavior could give us interesting information.

The quantification of monosaccharides by GC–MS of their trimethylsilyl-ester *O*-methyl glycosyl residues obtained after acidic methanolysis and derivatization reports the total content of soluble monosaccharides in the wine, which are constituents of both oligosaccharides (<5 kDa) and polysaccharides of greater molecular weight (>5 kDa). PRAG, RG-II, and MP polysaccharide families [29] are calculated from the content of these monosaccharides. Therefore, the content of each family informs us about the total content of the wine soluble fractions with molecular weights within the previously described range.

**Figure 2:**
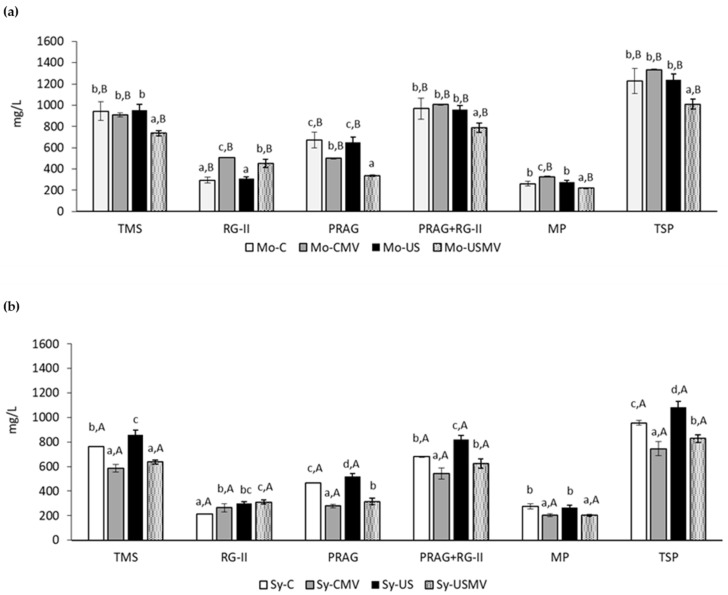
Total monosaccharide and polysaccharide families (mg/L) a in ‘Syrah’ (**a**) and ‘Monastrell’ (**b**) wines according to treatment. Average of the three measurements. Different letters indicate statistical differences (*p* < 0.05). Upper-case letters compare control wines. Lower-case letters compare wines with sonication treatment. TMS, total monosaccharides components of the precipitated polysaccharides; RG-II, rhamnogalacturonan type II; MP, mannoproteins; PRAG, polysaccharides rich in arabinose and galactose; TSP, total soluble polysaccharides.

Figure 2a,b show that control wines from ‘Monastrell’ presented higher content of PRAG, RG-II, and PRAG + RG-II pectic families than the control wines from ‘Syrah’, while the mannoprotein content was similar.

These differences between the control wines are related to the structural and compositional differences observed in the cell walls of the grapes of both varieties, especially the amount of cell wall material. Pérez-Porras et al. [3] describe the ‘Monastrell’ variety as having thicker cell walls and greater cell wall material than ‘Syrah’, therefore, grape varieties with greater cell material meant a higher content of polysaccharides in the wine. This higher concentration is interesting from a sensory point of view, since PRAG and RG-II modulate the astringency sensation of wines, since they reduce the interactions between wine tannins and saliva proteins [35].

The sonication of crushed ‘Syrah’ grapes facilitated the extraction of pectic polysaccharides from the grape cell walls to the wine. Results show that ultrasonic treatment favored the extraction of PRAG, RG-II, TMS, and TSP in ‘Syrah’ wines (Figure 1a), while it did not affect the MP content, which was similar to that of the control wine. MP are obtained, along with mannans and glucans, from the cell wall of yeast [36]; since alcoholic fermentation was developed after the application of US, the yeasts were not affected, and no differences in wines were observed.

However, ‘Monastrell’ wine made from sonicated grapes did not show significant differences in the content of PRAG, RG-II, TMS, TSP, and MP with the control wine, whereas large increases were observed with the same grapes when maceration lasted seven days [3]. Differences in composition of grape cell walls between grape varieties has been widely studied, as well as related to differences in the extractability of the compounds of interest [37,38]. Due to the high affinity of ‘Monastrell’ cell walls for phenolic compounds, higher than that of ‘Syrah’ and their high concentration, they could have bond part of the extracted phenolics, and precipitated afterwards, explaining the decrease in phenolics and the not-so-high concentration of polysaccharides in sonicated ‘Monastrell’ wines. It is clear that with longer maceration time and high quantity of cell walls available, the effect of ultrasounds became more evident, as shown in Pérez-Porras et al. [3], since the quantity of soluble polysaccharides arising for the degradation of high quantities of suspended cell wall material increased.

The use of the modified vinification changed the polysaccharide profile for both control wines and those made from sonicated grapes. As expected, the elimination of suspended cell wall material led to a decrease in the quantities of soluble polysaccharides. When the suspended material was eliminated, ‘Monastrell’ wines showed lower content of TMS (although differences were not significant for control wines), PRAGs, and, in the case of the wines made from sonicated grapes, lower TSP. Similar results were observed in ‘Syrah’ wines; however, the decreases in TMS, PRAG and TSP were much more important for ‘Syrah’ wines.

The family of polysaccharides that increased its concentration when the modified vinification was made was RG-II. Several authors [19,30,39,40,41] describe that pectic polysaccharide profile of most wines shows higher content of PRAG than RG-II, whereas this profile was different in wines made with the modified vinification (higher content of RG-II than PRAG). The RG-II molecule accumulated when pectolytic enzymes are used as previously observed [25], and the addition of enzymes during the settling step probably favored its presence.

## 4. Conclusions

We could demonstrate that the lack of effect of the sonication of some varieties, especially when skin maceration was short, was not only due to the rigidity and thickness of the cell structures of these grapes, but also to a high amount of suspended material originated from these thick cell walls and their high affinity for phenolic compounds. Our hypothesis was confirmed when the elimination of the suspended material led to a decrease in soluble polysaccharides (whose origin is the suspended cell wall material, mainly from pulp) in wines, but a great increase in color and phenolic compounds in ‘Monastrell’ sonicated grapes.

From a practical point of view, this modification in the vinification process is complicated for a winery, but maybe other enological practices that could limit the quantity of suspended material, such as the enzymes used for the clarification of musts, could be added to the process, such as pectin lyase; moreover, when this enzyme, as demonstrated by Osete-Alcaraz et al. [32], generates high quantities of low molecular polysaccharides that form soluble and stable combinations with phenolic compounds, helping to maintain wine quality with time.

## Figures and Tables

**Table 1:** Chromatic characteristics and phenolic compound composition of the different wines.

	CI	Hue	TPI	TA	PA	MCPT
‘Monastrell’						
Mo-C	10.31 ± 0.46 b	0.57 ± 0.01 a	52.11 ± 0.38 b	539.30 ± 14.00 c	23.52 ± 1.42 a	1121.51 ± 8.69 b
Mo-US	7.86 ± 0.55 a	0.73 ± 0.00 d	42.29 ± 2.13 a	358.17 ± 17.91 a	25.47 ± 1.75 a	932.91 ± 6.24 a
Mo-CMV	10.55 ± 0.16 b	0.65 ± 0.01 c	49.60 ± 0.37 b	451.39 ± 3.93 b	31.31 ± 0.43 b	1291.61 ± 0.85 c
Mo-USMV	12.37 ± 0.28 d	0.61 ± 0.01 b	58.49 ± 0.79 c	550.72 ± 15.06 c	32.21 ± 0.93 b	1532.82 ± 20.21 d
‘Syrah’						
Sy-C	19.40 ± 0.24 a	0.46 ± 0.01 b	58.77 ± 0.02 a	969.63 ± 12.57 a	40.76 ± 2.98 a	995.89 ± 26.27 a
Sy-US	23.47 ± 0.44 b	0.47 ± 0.00 b	70.91 ± 1.31 c	1087.59 ± 35.62 b	49.07 ± 0.80 bc	1340.05 ± 46.32 c
Sy-CMV	23.16 ± 0.34 b	0.44 ± 0.00 a	65.92 ± 0.08 b	1088.31 ± 23.33 b	45.31 ± 1.07 b	1103.79 ± 41.89 b
Sy-USMV	24.77 ± 0.60 c	0.46 ± 0.00 ab	75.65 ± 0.85 d	1114.39 ± 11.44 b	50.49 ± 1.18 c	1315.48 ± 0.85 c

Abbreviations: CI: color intensity, TA: total anthocyanins (mg/L), TPI: total polyphenol index, PA: polymeric anthocyanins (mg/L), MCPT: methylcellulose precipitable total tannins (mg/L). Different letters in the same column and for each type of wine mean statistically significant differences (*p* < 0.05) (n = 3 replicates for each variety).

**Table 2:** Tannin concentration and composition in the studied wines.

	TTp	mDP	%EGC	%Gal	EGC	ECG
‘Monastrell’						
Mo-C	752.70 ± 59.91 bc	6.82 ± 0.28 b	18.63 ± 0.59 c	2.10 ± 0.31 a	472.87 ± 28.47 b	53.17 ± 7.70 ab
Mo-US	534.19 ± 45.37 a	6.11 ± 0.08 a	16.68 ± 0.55 b	2.32 ± 0.04 ab	301.42 ± 35.46 a	41.92 ± 4.09 a
Mo-CMV	699.01 ± 37.28 b	8.05 ± 0.21 c	17.38 ± 0.82 bc	2.34 ± 0.09 ab	410.65 ± 40.84 b	55.17 ± 2.16 b
Mo-USMV	834.19 ± 13.09 c	6.82 ± 0.07 b	15.10 ± 0.06 a	2.65 ± 0.07 b	424.84 ± 8.50 b	74.53 ± 0.77 c
‘Syrah’						
Sy-C	440.57 ± 56.58 a	6.46 ± 0.29 b	22.60 ± 1.36 b	4.54 ± 0.49 a	332.56 ± 59.72 a	65.98 ± 4.13 a
Sy-US	759.78 ± 54.25 b	5.20 ± 0.09 a	19.58 ± 0.55 a	4.24 ± 0.09 a	496.94 ± 44.14 bc	107.40 ± 5.74 b
Sy-CMV	464.98 ± 97.36 a	6.59 ± 0.19 b	23.92 ± 0.37 b	4.17 ± 0.26 a	369.93 ± 72.59 ab	65.02 ± 16.13 a
Sy-USMV	843.46 ± 8.74 b	4.84 ± 0.07 a	18.29 ± 0.10 a	4.29 ± 0.15 a	515.23 ± 7.21 c	120.87 ± 4.76 b

Abbreviations: TTp: total tannins (mg/L) determined by the phloroglucinolysis method, mDP: mean degree of polymerization, %EGC: percentage of epigallocatechin, %Gal: percentage of galloylation, ECG: concentration of epicatechin gallate (μM), EGC: concentration of epigallocatechin (μM). Different letters in the same column and for each type of wine mean statistically significant differences (*p* < 0.05) (n = 3 biological replicates for each variety).

**Table 3:** Total area measured in the size exclusion chromatography analysis for the wine phenolic compounds and area corresponding to high molecular mass (HMM) (Fraction 1), medium molecular mass (MMM) (Fraction 2), and those corresponding to low molecular mass phenolic compounds (LMM) (Fraction 3).

	Total Area	Fraction 1 (HMM)	Fraction 2 (MMM)	Fraction 3 (LMM)
‘Monastrell’				
Mo-C	1.53	0.29 (19.03%)	0.51 (33.24%)	0.69 (45.40%)
Mo-US	1.27	0.26 (20.52%)	0.49 (38.27%)	0.49 (38.71%)
Mo-CMV	1.43	0.33 (23.09%)	0.51 (35.58%)	0.56 (38.91%)
Mo-USMV	1.74	0.46 (26.66%)	0.61 (34.70%)	0.63 (36.41%)
‘Syrah’				
Sy-C	1.66	0.17 (10.14%)	0.65 (39.04%)	0.83 (50.17%)
Sy-US	2.09	0.30 (14.40%)	0.86 (41.29%)	0.91 (43.60%)
Sy-CMV	1.86	0.21 (11.42%)	0.73 (39.12%)	0.91 (48.79%)
Sy-USMV	2.28	0.36 (15.94%)	0.91 (40.06%)	0.99 (43.34%)

The number in parenthesis indicates the percentage that every fraction means from the total area. F1: compounds with a molecular mass ranged between 840,000 and 30,000 g/mol, corresponding to compounds eluting from 10 to 12.2 min in the SEC analysis. F2: compounds with a molecular mass ranged between 30,000 and 1000 g/mol, corresponding to compounds eluting from 12.2 to 13.7 in the SEC analysis. F3: compounds with a molecular mass lower than 1000 g/mol and corresponding to compounds eluting from 13.7 to 16 min in the SEC analysis.

## Data Availability

The original contributions presented in the study are included in the article, further inquiries can be directed to the corresponding authors.
